# General psychopathology links burden of recent life events and psychotic symptoms in a network approach

**DOI:** 10.1038/s41537-020-00129-w

**Published:** 2020-12-15

**Authors:** Linda T. Betz, Nora Penzel, Lana Kambeitz-Ilankovic, Marlene Rosen, Katharine Chisholm, Alexandra Stainton, Theresa K. Haidl, Julian Wenzel, Alessandro Bertolino, Stefan Borgwardt, Paolo Brambilla, Rebekka Lencer, Eva Meisenzahl, Stephan Ruhrmann, Raimo K. R. Salokangas, Frauke Schultze-Lutter, Stephen J. Wood, Rachel Upthegrove, Nikolaos Koutsouleris, Joseph Kambeitz, the PRONIA consortium

**Affiliations:** 1 Department of Psychiatry and Psychotherapy, Faculty of Medicine and University Hospital of Cologne, University of Cologne, Cologne, Germany; 2 Department of Psychiatry and Psychotherapy, Ludwig-Maximilian-University, Munich, Germany; 3 Institute for Mental Health, University of Birmingham, Birmingham, UK; 4 Department of Psychology, Aston University, Birmingham, UK; 5 Orygen, Melbourne, VIC Australia; 6 Centre for Youth Mental Health, University of Melbourne, Melbourne, VIC Australia; 7 Department of Neurological and Psychiatric Sciences, University of Bari, Bari, Italy; 8 Department of Psychiatry (UPK), University of Basel, Basel, Switzerland; 9 Department of Psychiatry and Psychotherapy, University of Lübeck, Lübeck, Germany; 10 Department of Neurosciences and Mental Health, Fondazione IRCCS Ca’ Granda Ospedale Maggiore Policlinico, University of Milan, Milan, Italy; 11 Department of Psychiatry, University of Münster, Münster, Germany; 12 Otto Creutzfeldt Center for Behavioral and Cognitive Neuroscience, University of Münster, Münster, Germany; 13 Department of Psychiatry and Psychotherapy, Medical Faculty, Heinrich-Heine University, Düsseldorf, Germany; 14 Department of Psychiatry, University of Turku, Turku, Finland; 15 Department of Psychology and Mental Health, Faculty of Psychology, Airlangga University, Surabaya, Indonesia; 16 Max-Planck Institute of Psychiatry, Munich, Germany; 17 Institute of Psychiatry, Psychology and Neuroscience, King’s College London, London, UK

**Keywords:** Psychosis, Human behaviour

## Abstract

Recent life events have been implicated in the onset and progression of psychosis. However, psychological processes that account for the association are yet to be fully understood. Using a network approach, we aimed to identify pathways linking recent life events and symptoms observed in psychosis. Based on previous literature, we hypothesized that general symptoms would mediate between recent life events and psychotic symptoms. We analyzed baseline data of patients at clinical high risk for psychosis and with recent-onset psychosis (*n* = 547) from the Personalised Prognostic Tools for Early Psychosis Management (PRONIA) study. In a network analysis, we modeled links between the burden of recent life events and all individual symptoms of the Positive and Negative Syndrome Scale before and after controlling for childhood trauma. To investigate the longitudinal associations between burden of recent life events and symptoms, we analyzed multiwave panel data from seven timepoints up to month 18. Corroborating our hypothesis, burden of recent life events was connected to positive and negative symptoms through general psychopathology, specifically depression, guilt feelings, anxiety and tension, even after controlling for childhood trauma. Longitudinal modeling indicated that on average, burden of recent life events preceded general psychopathology in the individual. In line with the theory of an affective pathway to psychosis, recent life events may lead to psychotic symptoms via heightened emotional distress. Life events may be one driving force of unspecific, general psychopathology described as characteristic of early phases of the psychosis spectrum, offering promising avenues for interventions.

## Introduction

Stressful life events, such as losing a loved one, failure in an exam or change of residence, have been repeatedly linked to the onset, course and outcome of psychotic disorders^1–7^. Specifically, prior research has documented associations between recent life events and broad outcome categories, such as diagnosis of a psychotic disorder and compound measures of positive symptomatology^2,7,8^. However, the specific pathways linking recent stressful life events and expression of psychotic symptomatology in the psychosis spectrum, including early stages, i.e. at-risk stages and recent-onset psychosis, are yet to be fully understood^2,3,7,9,10^.

Recent years have seen the emergence of two distinct trends in the fields of psychopathology and psychiatry that may help to address this issue. First, there is a growing awareness that domains of affective, cognitive and negative symptoms need to be considered to gain a thorough understanding of the etiology of psychosis^11–17^. Second, vital insight can be acquired when modelling psychosis via individual symptoms instead of diagnostic cut-offs and sum scores of symptoms^18^. Specifically, symptom networks may constitute an insightful way to conceive the complex dependencies between life events and symptoms in early phases of the psychosis spectrum^12,18,19^. Here, mental disorders are conceptualized as sets of interacting symptoms that show specific associations with other clinically relevant factors, such as stressful recent life events^12,19^.

Adopting a network-based perspective on psychopathology allows the identification of potential psychological pathways from adverse events to psychotic symptoms^12,18,19^. For example, in a previous network analysis, childhood trauma was found to connect to positive and negative symptoms only via symptoms of general psychopathology^12^. These findings suggest that adverse events might result in psychosis through heightened emotional reactivity to stress and add to the accumulating evidence for an affective pathway to psychosis^13,20^. Corroborating this idea, recent findings suggest that the association between a range of lifetime traumatic events and psychotic-like experiences is largely mediated by general psychopathology in a sample of prisoners^21^. Overall, it seems reasonable to hypothesize that recent stressful life events may predispose expression of psychotic symptomatology by a pathway similar to childhood trauma, i.e. via heightened emotional distress^13,20,22^. Childhood trauma may interact with adult life events by sensitizing individuals to future stressful events and by increasing the risk of experiencing later burdensome life events^23,24^.

Inherently, this reflects a within-person process: individual burden following life events is paralleled by an increase in an individual’s emotional distress. Network models estimated from cross-sectional data, though valuable for deriving unique associations in high-dimensional variable spaces, do not necessarily reflect within-person dynamics over time^25^. Rather, cross-sectional networks reflect combined influences at both the within-person and the between-person level^26^. Panel data, in which many subjects are measured at multiple times, allow the examination of average within-person dynamics, i.e. temporal dependencies. To date, this possibility has not been exploited in research on the association between life events and psychopathology observed in the early psychosis spectrum.

Additionally, given large interindividual differences in the experience of the same life events, studying the subjective burden of recently experienced life events rather than the mere exposure may be most insightful^27–29^. Individuals may, for instance, perceive a given life event as less burdensome following prior exposure that allowed them to develop adaptive strategies to deal with similar future adversity^30^. Likewise, life events that are commonly perceived as positive and little stressful may evoke burden in certain individuals. Ideally, analyses should therefore not be limited to a predefined set of negative or traumatic recent life events.

In the current study, we use network analysis to investigate pathways between the cumulative burden of a comprehensive set of recent life events and individual positive, negative and general symptoms in the early psychosis spectrum. Based on previous literature^12,21,31^, we hypothesize that burden of recent life events will not be connected to positive and negative symptoms directly, but indirectly via general symptoms. In a control analysis, we examine whether childhood trauma can explain links between burden of recent life events and symptoms. Additionally, we investigate the dynamic, within-person interplay between burden of recent life events and symptomatology over time by using multiwave panel data.

## Results

### Sample characteristics

The final sample (*N* = 547) comprised 265 patients at clinical high risk for psychosis (CHR) and 282 patients with recent-onset psychosis (ROP). Overall, 47.3% of the participants were women and the average age was 24.7 years (SD = 5.6). On average, 0.3% of the baseline network variables were missing. ROP participants were significantly older and comprised more men as compared to CHR participants. Significant differences were also present in symptomatology and functioning (Table 1). Prevalence of SCID-diagnoses in the sample are available in Supplementary Table 1. Reported number of life events and mean burden did not differ between the groups. For a comparison of demographic and clinical variables in women and men, see Supplementary Table 2. The three most common life events in our sample were “significant negative incident related to partnership”, “major examination successful”, and “removal from living place” (for an overview of reported life events in the sample, Supplementary Fig. 1). In the longitudinal analysis, 337 participants (168 CHR, 169 ROP) were included. This sample did not differ significantly in most demographic and clinical characteristics at baseline from participants excluded due to missing data (Supplementary Table 3).

**Table 1:** Demographic and clinical characteristics of the sample at baseline.

Variable	CHR (*n* = 265)	ROP (*n* = 282)	Whole sample (*n* = 547)	Comparison (CHR vs. ROP)
Sex (% female)	52.7	42.7	47.5	χ^2^ = 5.40, *p* = 0.020
Age	23.6 (5.2)	25.6 (5.9)	24.7 (5.6)	Z = −4.08, *p* < 0.001
PANSS (subscale scores)				
Positive	11.2 (3.6)	18.5 (6.1)	15.0 (6.2)	Z = −13.7, *p* < 0.001
Negative	13.6 (6.6)	16.1 (7.6)	14.9 (7.2)	Z = −4.09, *p* < 0.001
General	29.3 (8.1)	34.7 (10.7)	32.1 (9.9)	Z = −6.39, *p* < 0.001
Total	54.1 (15.4)	69.3 (20.4)	62.0 (19.7)	Z = −9.02, *p* < 0.001
Number of recent life events (median, range)	3 (0–10)	3 (0–10)	3 (0–10)	Z = 0.63, *p* = 0.532
Burden of recent life events	6.6 (6.2)	6.4 (6.6)	6.5 (6.4)	Z = 0.32, *p* = 0.748
CTQ-SF (subscale scores)				
Emotional abuse	10.4 (4.5)	9.7 (4.4)	10.0 (4.5)	Z = 1.65, *p* = 0.101
Physical abuse	6.5 (2.9)	6.5 (3.1)	6.5 (3.0)	Z = −0.19, *p* = 0.857
Sexual abuse	6.0 (2.7)	6.1 (3.0)	6.1 (2.9)	Z = −0.68, *p* = 0.504
Emotional neglect	11.9 (4.0)	11.4 (4.1)	11.7 (4.1)	Z = 1.17, *p* = 0.255
Physical neglect	7.4 (2.6)	7.6 (3.0)	7.5 (2.8)	Z = −0.71, *p* = 0.477
GAF-disability (past month)	52.3 (13.0)	45.0 (14.1)	48.6 (14.1)	Z = 6.06, *p* < 0.001
GAF-symptoms (past month)	52.1 (11.3)	41.0 (14.3)	46.4 (14.1)	Z = 9.22, *p* < 0.001
BDI-II (total score)	26.3 (12.2)	21.6 (13.1)	23.9 (12.9)	Z = 3.95, *p* < 0.001

Means (SD) unless stated otherwise.

*BDI* Beck Depression Inventory, *CHR* clinical high risk, *CTQ-SF* Childhood Trauma Scale-Short Form, *GAF* global assessment of functioning, *PANSS* Positive and Negative Syndrome Scale, *ROP* recent-onset psychosis.

### Network analysis

**Fig. 1:**
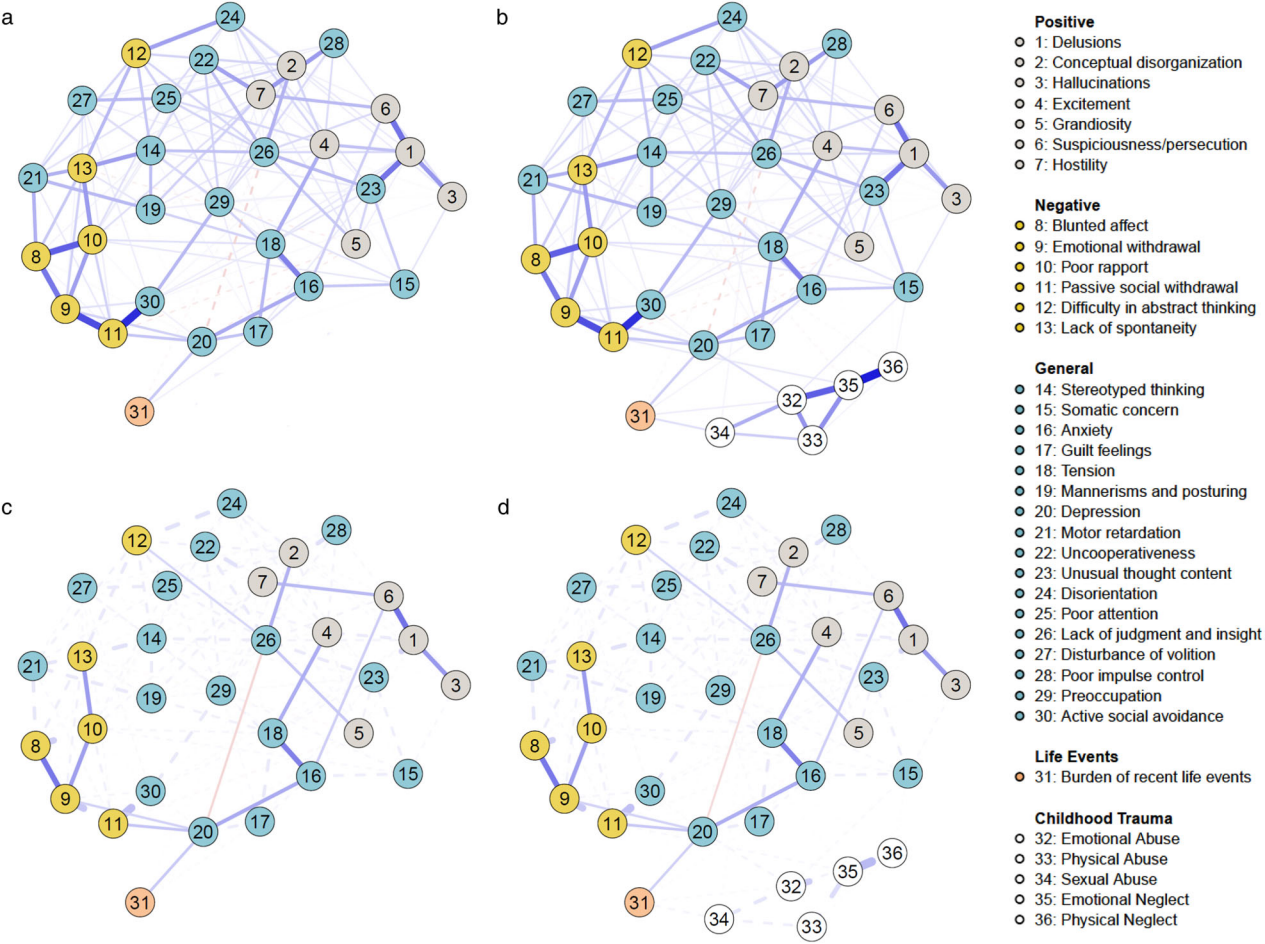
Cross-sectional network of relationships between burden of recent life events and symptomatology assessed with the Positive and Negative Syndrome Scale (PANSS^34^) in the early psychosis spectrum (*n* = 547). Upper panel: Network depicting unique associations between burden of recent life events and individual symptoms. **a** before and **b** after controlling for different childhood trauma types as covariates. The wider the edge, the stronger the association. Blue (red) edges reflect positive (negative) connections. Lower panel: Network highlighting shortest paths between burden of recent life events and the positive and negative symptom domain of the PANSS. **c** before and **d** after controlling for different childhood trauma types as covariates. Solid lines represent shortest paths, dashed lines represent connections that do not lie on the shortest paths. The wider the edge, the stronger the association. Blue (red) edges reflect positive (negative) connections.

Figure 1a illustrates the L_1_-regularized Gaussian graphical model^32,33^, i.e. the regularized, undirected network of partial correlation coefficients between individual items of the Positive and Negative Syndrome Scale (PANSS^34^) and the cumulative burden of reported recent life events we estimated from the data. Of 465 possible edges, 177 were retained in the L_1_-regularized partial correlation network. We identified positive relationships between burden of recent life events (node 31) and the PANSS items depression (node 20) as well as guilt feelings (node 17). Additionally, there was a small negative association between burden of recent life events (node 31) and lack of judgment and insight (node 26). The shortest paths (Fig. 1c) display the shortest routes that connect burden of recent life events (node 31) to each individual positive and negative symptom of the PANSS (nodes 1–13). The shortest route to reach most positive psychotic symptoms from burden of recent life events is via depression (node 20) and anxiety (node 16). Specifically, anxiety links to suspiciousness/persecution (node 6), which, in turn, connects to delusions (node 1), hallucinations (node 3) and hostility (node 7). Excitement (node 4) is reached by burden of recent life events via a path through depression (node 20) and tension (node 18). Even though the path from burden of recent life events via depression (node 20) and lack of judgment/insight (node 26) per definition constitutes the shortest route to positive symptoms conceptual disorganization (node 2) and grandiosity (node 5), the negative association between depression (node 20) and lack of judgment/insight (node 26) “disrupts” these pathways. Conversely, an extended pathway to conceptual disorganization (node 2) and grandiosity (node 5) via excitement (node 4), features positive connections only. All negative symptoms in the network can be reached via depression (node 20). Robustness analyses showed that the network was very stable and identified edges were estimated with good accuracy (Supplementary Results 1 and Supplementary Figs 2 and 3).

When corrected for the influence of different types of childhood trauma, the major pathways from burden of recent life events to positive and negative symptoms via general psychopathology remain unaffected (Fig. 1b, d). In the network, emotional and sexual abuse are positively linked to burden of recent life events. Types of childhood trauma also show several independent pathways to psychotic symptoms via general psychopathology, such as from emotional abuse via depression and anxiety to suspiciousness, and from emotional abuse via somatic concern to hallucinations, grandiose ideas and delusions. Robustness analyses showed that the network was very stable and identified edges were estimated with good accuracy (Supplementary Results 1 and Supplementary Figs 4 and 5). Networks of CHR and ROP participants differed significantly neither in network structure, global strength of connections, nor strength of any individual connections (Supplementary Results 2 and Supplementary Fig. 6). Similarly, there were no significant differences between the networks estimated separately in women and men (Supplementary Results 3 and Supplementary Fig. 7).

### Exploring the longitudinal relationship between burden of recent life events, depression and guilt feelings

**Fig. 2:**
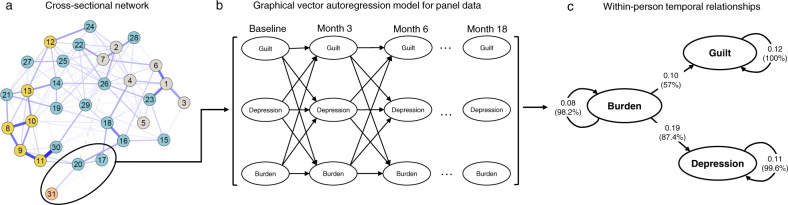
Longitudinal analysis of the relationship between burden of recent life events, depression and guilt feelings in the early psychosis spectrum (*n* = 337). **a** Focusing on symptoms that showed connections with burden of recent life events in the cross-sectional network, modeled using 30 PANSS items and burden of recent life events, **b** up to seven equidistant, consecutive measurement occasions, each about three months apart, were used for modeling **c** the longitudinal relationships between burden of recent life events, depression, and guilt in a graphical vector autoregression model for panel data (*panelgvar*). Parameters reflecting the longitudinal relationships were standardized to partial directed correlations. All depicted parameters estimates were significant (*p* < 0.001). The percentages in brackets indicate how often each edge was included across 1000 bootstrap models.

Figure 2 shows the temporal effects, standardized to partial directed correlations, between burden of recent life events, depression and guilt feelings obtained from a graphical vector autoregression model for panel data^25^. There were positive directed associations from burden of recent life events to both depression (*β* = 0.19, *p* < 0.001) and from burden to guilt feelings (*β* = 0.10, *p* = 0.002), but not vice versa. This finding suggests that when an individual experiences higher levels of burden at one timepoint, levels of depression and guilt feeling are increased at the follow-up timepoint. Autoregressive effects for each variable were as follows: burden of recent life events = 0.08 (*p* = 0.034), depression = 0.11 (*p* < 0.001), guilt = 0.12 (*p* < 0.001), i.e. the amount of within-person carry-over effect from one timepoint to the next was about equally large for all three variables. This implies that timepoints on which a patient scored above his or her expected score are likely to be followed by timepoints on which he or she still scores above the expected score again, and vice versa. The bootstrapping analysis showed that all estimated edges were included in the majority of estimated models, suggesting a general robustness of the results to sampling variation.

## Discussion

In the present study, we investigated the relationship between the burden of recent life events and specific symptoms in the early psychosis spectrum. Specifically, we conducted a cross-sectional network analysis including all individual symptoms of the PANSS (positive, negative and general psychopathology symptoms) and cumulative burden of recent life events. Our results show that burden of recent life events is not directly linked to any of the positive or negative symptoms. As hypothesized, shortest pathways in the network illustrate that burden of recent life events is connected to positive and negative symptoms only via general psychopathology such as depression, guilt feelings, anxiety or tension. Importantly, these results were robust with respect to the inclusion of different types of childhood trauma. Overall, this suggests that general psychopathology symptoms mediate the relationship between life event burden and expression of psychotic symptomatology. Further, we used longitudinal modelling based on panel data to identify the temporal relationship between burden of recent life events and symptoms. Here, burden predicted depression and guilt feelings 3 months later, suggesting a specific effect of life event burden on the severity of affective symptomatology at the cross-sectional and within-person level. In summary, these findings extend previous cross-sectional evidence obtained in a sample of prisoners^21^ to a clinical sample of patients in the early psychosis spectrum, including a more comprehensive set of life events and symptoms, and additionally provide a nuanced longitudinal analysis that suggested temporal priority of burden of recent life events over affective symptoms at the level of the individual.

Our results can be interpreted in light of an affective pathway to psychosis. According to this hypothesis, adverse life events lead to expression of psychotic symptomatology through heightened emotional distress^13,20,22^. Major burdensome life events result in negative affect—e.g. in the form of depression, anxiety, guilt feelings and tension, as indicated previously^6,21^ and by our analysis. This in turn may increase sensitivity to minor daily hassles, potentially facilitating the development of psychotic symptomatology^20,35^. Similarly, previous work suggests that early adverse events, such as childhood trauma, may trigger a pathway to psychotic symptoms via general psychopathology, in particular anxiety, tension and depression^12,36,37^. We could replicate several of these previously identified pathways^12^ in our control analysis, such as the pathway from abuse via depression and anxiety to suspiciousness, and the pathway from abuse via somatic concern to a cluster of hallucinations and delusions. One putative biological mechanism underlying this increased stress reactivity may involve alterations in the hypothalamus-pituitary-adrenal axis, which may subsequently give rise to psychotic symptoms via increased dopamine receptor densities and dopamine release^20,38^. Complementarily, heightened emotional distress can also be understood in terms of cognitive models of psychosis: Emotional changes following burdensome life events, such as depression, guilt feelings, anxiety and tension, may feed back into moment-by-moment processing of paranoid ideas and anomalous experiences and make their occurrence more likely^10^.

Another important finding of our analysis is that early adverse experiences and burden of recent life events are not independent: experience of childhood abuse makes the experience of life events as burdensome more likely. This could be due to a lasting vulnerability to stress following childhood trauma, characterized by an enhanced experience of life events to be burdensome and stressful^10,20,23,39^. Likewise, a personal environment associated with adverse childhood experiences might also entail more conflicts, and thus, more burdensome life events, in adolescence and early adulthood. Importantly, our results suggest that early and recent stressful life events have similar, yet independent effects on psychotic symptoms, as pathways from recent life events to psychotic symptoms via general psychopathology were present in the network even after inclusion of childhood trauma. The interplay between early and recent stressful life events underscores the relevance of analyzing the association between risk factors to advance the understanding of the etiology of psychotic symptomatology^18,22^. More research is necessary to work out differences in the specific mechanisms of actions of recent stressful life events and childhood trauma on general psychopathology and psychotic symptoms.

Overall, our results suggest that life events may be one driving force of unspecific, general psychopathology described as characteristic of early phases of mental disorders, corroborating previous considerations^11,14,15,19,31^. Early transdiagnostic pathways from life events to general psychopathology also align with the idea of multifinality in the emergence of psychopathology following recent stressful life events, similar to early trauma^12,22,39,40^. Accordingly, life events have been associated not only with increased risk for psychosis, but also with onset and course of other disorders, such as depressive^41,42^, anxiety^43^, bipolar^44,45^ or obsessive-compulsive disorder^46^. In the network view, life events trigger negative affect, from which further activity may then “spread” in the network. Yet, it is still unclear when this is the case and transdiagnostic approaches might help to shed light on this question^47^. In later stages of mental illness, symptoms may then sustain each other even after cessation of external stressors such as life events^19^. From a clinical perspective, this underscores a growing consensus that general psychopathology symptoms should receive more attention in the management of patients with suspected and early psychosis^10–12,14,15^, in particular given burdensome personal circumstances or affective dysregulation in early stages of psychotic disorder. One viable strategy may involve reducing certain types of burdensome life events, such as conflicts in the family or partnership, through appropriate interventions, e.g. assertive community treatment or family-focused therapy, to reduce stress in the social environment of patients^20,31,48,49^.

Several limitations regarding the present results need to be taken into consideration. First, the comprehensive main network was built on cross-sectional data, allowing no conclusion about temporal priority and relationships in the individual. We aimed to clarify the directionality of the most important connections and examined averaged within-person processes by providing an additional longitudinal analysis based on panel data. Due to modeling constraints, selection of items used in this analysis was based on the connections in the more comprehensive cross-sectional network, which may not be representative of the most important longitudinal relationships. Second, we used the PANSS to assess symptomatology in an early psychosis spectrum sample including patients with ROP and CHR. It might be argued that alternative scales are more appropriate for assessment in CHR; however, we opted for the PANSS over other tools designed specifically for CHR populations as it covers a broader range of symptomatology, and generally shows good construct and convergent validity also in CHR samples^50^. Third, as sample sizes were small relative to the number of nodes in the network, statistical power in the comparison of networks of CHR and ROP, as well as those estimated separately in women and men, may have been insufficient to detect relevant differences. Larger sample sizes are likely needed to gain a better understanding of the role of life events in different stages of the psychosis spectrum. Larger sample sizes would also enable investigations of the role of specific types of life events as well as analyses in subgroups, such as affective and nonaffective psychosis, in which life events might exert different effects. Lastly, the group-level design of our analysis, focused on burden of recent life events as a generalized measure, does not allow direct conclusions for individual patients nor individual types of life events. We also implicitly assume that experiencing no life events is equivalent to experiencing life events without perceiving concomitant burden. Future studies may assess the impact of specific life events in psychosis by means of extensive longitudinal data collected in the individual, e.g. experience sampling methods (ESM). By following a group of patients longitudinally, with repeated ESM assessments, such a study design would allow to examine how life events alter the interplay between emotional reactivity to daily stressors and symptomatology at the level of the individual.

In sum, we adopted a network perspective to investigate the relationship between burden of recent life events and a comprehensive set of symptoms in a sample of patients at risk for psychosis and with recent-onset psychosis. Our findings provide further evidence for an affective pathway to psychosis^12,14,21,22^ and show that unspecific, general psychopathology mediates the association between life event burden and expression of psychotic symptomatology, suggesting promising avenues for targeted interventions. These results highlight the added value of network analysis in deriving insights into psychological pathways implicated in the complex etiology of psychotic symptoms.

## Methods

### Participants

We analyzed data from participants at CHR (*n* = 275) and patients with ROP (*n* = 316) of the multicentric Personalized Prognostic Tools for Early Psychosis Management study (PRONIA, https://www.pronia.eu; German Clinical Trials Register identifier DRKS00005042)^51^. Participants aged 15–40 were recruited between February 2014 and December 2017 in 10 academic early-recognition services in five European countries, i.e. Finland, Germany, Italy, Switzerland and the United Kingdom. The scheduled total follow-up period was 18 months, during which participants were assessed every three months. For the longitudinal analyses, we used data of up to month 18 past study inclusion, leading to a possible maximum of seven approximately equidistant measurement occasions for each participant. We included participants with available information on life events and the PANSS at the baseline assessment, yielding a final sample size of *N* = 547 (*n* = 265 CHR participants, *n* = 282 ROP participants). For longitudinal modeling, we used a subset of these 547 participants who had data on at least two consecutive measurement occasions available (*n* = 337). Details on inclusion and exclusion criteria have been published previously^51^. In short, the CHR state in PRONIA was defined by: (1) cognitive disturbances (COGDIS), as assessed by the Schizophrenia Proneness Instrument (SPI-A^52^); and/or (2) adapted PRONIA ultra-high-risk (UHR) criteria for psychosis, as measured by the Structured Interview for Psychosis-Risk Syndromes (SIPS^53^). Specific exclusion criteria for CHR individuals were (1) intake of antipsychotic medication for more than 30 cumulative days at or above the minimum dosage threshold defined by the DGPPN S3 Guidelines for the treatment of first-episode psychosis (https://www.dgppn.de/_Resources/Persistent/43ca38d4b003b8150b856df48211df68e412d9c9/038-009k_S3_Schizophrenie_2019-03.pdf), and (2) any intake of antipsychotic drugs within the past 3 months before psychopathological baseline assessments at or above the minimum dosage threshold. To ensure that risk symptoms were not due to drug consumption, participants had to be abstinent from illegal drugs for at least 4 weeks prior to study entry.

For ROP patients, specific inclusion criteria included meeting full DSM-IV criteria for an affective or nonaffective psychotic episode in the past three months and first onset of psychosis during the last 24 months. ROP patients were excluded if they had taken antipsychotic medication for more than 90 days (cumulative number of days) at or above minimum dosage of the first-episode psychosis range of DGPPN S3 Guidelines.

All adult participants provided their written informed consent prior to study inclusion, and minor participants (defined as those younger than 18 years) provided written informed assent and their guardians written informed consent. The authors assert that all procedures contributing to this work comply with the ethical standards of the relevant national and institutional committees on human experimentation and with the Helsinki Declaration of 1975, as revised in 2008. The local research ethics committees at each study site approved the study.

### Burden of recent life events

Life events were recorded with the Cologne Chart of Life Events (CoLE^54^). The CoLE was adapted from the Munich Life Event List^55^ and comprises a list with 117 events from 12 domains (Supplementary Fig. 8). The interviewer asks the participant whether he or she experienced any event from these 12 domains in the last 12 months (baseline assessment) or since the least visit (at follow-up visits). For all reported events, the interviewer assigns the event to the most representative category from the list and notes duration and the participant’s subjective evaluation, experienced burden and controllability of each reported life event. For each measurement occasion, up to ten life events are recorded. For the present analyses, we focused on the burden of a given life event, which was scored on a 5-point Likert scale (0: no burden; 1: little burden; 2: moderate burden; 3: much burden; 4: very much burden). We computed the total burden of all reported life events by summing the individual burden ratings of each reported life event (maximum possible score = 40), excluding life events directly linked to the mental health status of the participants, such as hospitalization and start of psychopharmacological treatment, as these events are not commonly conceptualized as life events^56^. If a patient reported no life events, the total burden of recent life events was defined as 0.

### Symptomatology

For the present analysis, we used the 30 individual items from the PANSS^34^, which is a widely used, clinician-administered assessment of psychopathology typically associated with psychotic syndromes, with each item scored on a 7-point Likert severity scale from 1 (absent) to 7 (extreme). The reference period for the symptoms were the last 7 days.

### Covariates: domains of childhood trauma

As covariates in a separate control analysis, we included five domains of childhood trauma, assessed by the subscales of the Childhood Trauma Questionnaire—Short Form (CTQ-SF^57^), i.e. emotional neglect, physical neglect, emotional abuse, physical abuse and sexual abuse.

### Data analytic strategy

We conducted all analyses in the *R* language for statistical computing, version 3.6.3^58^. Throughout, we considered a significance level of α < 0.05, two-sided. Group comparisons for descriptive statistics were based on permutation-tests implemented in the *R* package ‘coin’, version 1.3-1^59^.

### Network estimation

We fitted a Gaussian graphical model in the form of a L_1_-regularized partial correlation network to the data^32,33^. Each node in the network corresponds to one of the included PANSS items and the burden of life events. Connections between nodes reflect the partial correlation (or, equivalently, conditional dependence relation) between these items and represent the strength of the association between two items after controlling for all other variables under consideration. To account for the ordinal nature of the network items, we computed the partial correlation matrix based on Spearman’s correlation coefficient. We recovered the optimal network by minimizing the extended Bayesian Information Criterion (EBIC) of a set of 100 networks estimated with the graphical lasso (glasso) algorithm that imposes L_1_-regularization^60,61^. L_1_-regularization ensures that small and likely spurious edges are removed from the model, leading to sparse, interpretable networks^32^. The EBIC itself has a hyperparameter that we set to 0 for the present analyses. For plotting both networks, we used a force-directed layout generated by the Fruchterman–Reingold algorithm based on the network including the covariates^62^. Additionally, we highlighted the shortest paths between the burden of life events variable and the positive and negative symptoms of the PANSS. The shortest path between two nodes represents the minimum number of steps necessary to go from one node to the other, highlighting possible pathways and mediators between life events and positive and negative symptoms^12,63^. We calculated the shortest pathways using Dijkstra’s algorithm^64^. We repeated all network estimation and visualization steps in a control analysis where we included the five domains of the CTQ (i.e. emotional abuse, physical abuse, sexual abuse, emotional neglect and physical neglect)^57^.

Additionally, we estimated networks separately for participants with CHR and ROP, and compared the resulting network structures statistically with a permutation test (1000 permutations)^65^ to formally assess whether networks of CHR and ROP participants differed from each other in (1) their network structures (i.e. the maximum of element-wise, absolute differences in edge weights), (2) global strength (i.e. the sum of all absolute edge weights) and (3) individual edge weights^65^. Due to the focus of the analysis, we restricted the comparison of individual edges to edges associated with burden of recent life events. Here, we corrected for multiple comparisons by controlling the false discovery rate^66^. Analogously, separate networks were estimated and compared for women and men (using combined data from CHR and ROP participants).

Network estimation and visualization steps were performed using the *R* package ‘qgraph’, version 1.6.5^67^ and statistical network comparison was conducted with the *R* package ‘NetworkComparisonTest’, version 2.2.1^65^.

### Robustness analyses

As recommended, we conducted several follow-up bootstrapping analyses on the calculated networks to investigate their proneness to sampling variation and stability under case-dropping using the *R* package ‘bootnet’, version 1.4^32,68^. These analyses (a) show how accurately the edges in the network are estimated by constructing a 95% bootstrapped confidence interval (CI) around them, and (b) indicate how stable edges and centrality indices are estimated via the centrality-stability (CS) coefficient^69^. This coefficient indicates the maximum proportion of observations that can be dropped while confidently (95%) retaining results that correlate highly (*r* > 0.7) with the results obtained in the original sample. A CS coefficient of 0.25 or above indicates adequate stability and a coefficient of 0.50 or above indicates high stability^32^. For all robustness analyses, we used 1000 bootstrap samples.

### Longitudinal relationship between burden of recent life events and symptoms

We explored longitudinal network relationships between burden of recent life events and symptoms connected to burden of recent life events in the baseline network by using a graphical vector autoregression model for panel data (panelgvar)^25^. The panelgvar-model allows to determine how these variables influence each other across the seven possible measurement occasions at the within-person, state-like level, while controlling for trait-like, between-person differences through the inclusion of a random intercept^26^. The panelgvar-model constrains the effects that variables have on each other to remain stable over the seven possible measurement occasions. In our analyses, we first fit a panelgvar*-*model in which all edges were included, using full information maximum likelihood estimation (FIML). Cases with missing observations are retained and the FIML estimation adjusts the likelihood function so that each participant contributes information on the variables that are observed. Second, we used a stepwise model search to find the model with optimal Bayesian information criterion (BIC), thresholding at α = 0.05 for the addition or pruning of individual edges. In the optimal model, no edge can be added or pruned to improve fit. The resulting temporal network encodes directed predictive effects between the variables over time, which reflect the within-person temporal relationships of the average participant^25^ (Fig. 2). A comprehensive explanation of the model goes beyond the scope of the present work (see the recent methodological article^25^ for details). We tested the robustness of the results to sampling variation by assessing how often each edge was included across 1000 bootstrapped models. All analyses were run with the *R* package ‘psychonetrics’, version 0.7.1.

### Reporting summary

Further information on research design is available in the Nature Research Reporting Summary linked to this article.

## Supplementary information

Supplementary Material

Reporting Summary Checklist

## Data Availability

Supplemental findings supporting this study are available on request from the corresponding author (LTB). The data are not publicly available due to Institutional Review Board restrictions—since the participants did not consent to their data being publicly available.

## Code availability

Code used for data analysis is available at: https://github.com/LindaBetz/Network_Life_Events_Psychosis.
