# Supplementary material for: General psychopathology links burden of recent life events and psychotic symptoms in a network approach
Source: NPJ Schizophr. 2020 Dec 15;6:40. doi: 10.1038/s41537-020-00129-w (PMC7738498; doi:10.1038/s41537-020-00129-w)
Supplement: Supplementary file 1 — Supplementary Material [file 41537_2020_129_MOESM1_ESM.pdf]

Supplementary Material for
"General Psychopathology Links Recent Life Events and Psychosis in a Network Approach"

Betz LT, Penzel N. et al.

Supplementary Table 1. Diagnosis ascertained by the Structured Clinical Interview for DSM-IV (SCID) in the Clinical High-Risk (CHR) and Recent Onset Psychosis (ROP) sample.

Supplementary Table 2. Comparison of baseline demographic and clinical characteristics of women and men.

Supplementary Table 3. Comparison of baseline demographic and clinical characteristics of those participants included in longitudinal modeling and those participants excluded due to missing data.

Supplementary Results 1. Robustness analyses.

Supplementary Results 2. Comparison of networks estimated in CHR and ROP.

Supplementary Results 3. Comparison of networks estimated in women and men.

Supplementary Figure 1. Life events in the early psychosis spectrum reported at baseline (*N* = 547).

Supplementary Figure 2. Edge values with 95% confidence intervals obtained from bootstrapping in the original sample for the main network model.

Supplementary Figure 3. Case-dropping bootstrap for the main network model.

Supplementary Figure 4. Edge values with 95% confidence intervals obtained from bootstrapping in the original sample for the main network model after inclusion of different childhood trauma types as covariates.

Supplementary Figure 5. Case-dropping bootstrap for the main network model after inclusion of different childhood trauma types as covariates.

Supplementary Figure 6. Cross-sectional networks of relationships between burden of recent life events and symptomatology assessed with the Positive and Negative Syndrome Scale (PANSS) estimated separately in Clinical High-Risk (CHR) and Recent Onset Psychosis (ROP) participants.

Supplementary Figure 7. Cross-sectional networks of relationships between burden of recent life events and symptomatology assessed with the Positive and Negative Syndrome Scale (PANSS) estimated separately in women and men.

Supplementary Figure 8. The Cologne Chart of Life Events.

PRONIA consortium members.

Supplementary Tables

Supplementary Table 1. Diagnosis ascertained by the Structured Clinical Interview for DSM-IV (SCID) in the Clinical High-Risk (CHR) and Recent Onset Psychosis (ROP) sample.

Diagnosis	Frequency (%)
**CHR (n = 265)**	
Major depressive disorder	51.3
No current axis I disorder	22.3
Obsessive compulsive disorder	3.8
Panic disorder	3.8
Generalized anxiety disorder	3.0
Adjustment disorder	1.9
Dysthymic disorder	1.9
Anxiety disorder NOS	1.5
Bipolar II disorder	1.5
Depressive disorder NOS	1.5
Cannabis dependence	1.1
Dissociative disorder	1.1
Social phobia	1.1
Bipolar I disorder	0.08
Other axis I disorder	0.08
Specific phobia	0.08
Anorexia	0.08
Bipolar disorder other	0.08
Body dysmorphic disorder	0.08
Somatization disorder	0.08
**ROP (n = 282)**	
Schizophrenia	36.9
Psychotic disorder NOS	14.5
Schizophreniform disorder	12.8
Brief psychotic disorder	8.2
Schizoaffective disorder	8.2
Major depressive disorder (with psychotic features)	7.4
Delusional disorder	6.7
Bipolar I disorder (with psychotic features)	5.0
Bipolar II disorder (with psychotic features)	0.04

Abbreviations: CHR: Clinical High-Risk; NOS: Not Otherwise Specified; ROP: Recent Onset Psychosis.

Supplementary Table 2. Comparison of baseline demographic and clinical characteristics of women and men. Means (SD) unless stated otherwise.

Variable	Women (n = 260)	Men (n = 287)	Comparison
Studygroup (% ROP)	46.2	56.4	$\chi^2 = 5.79$, $p = .020$
Age	24.8 (5.9)	24.5 (5.4)	$Z = -0.50$, $p = .617$
PANSS (subscale scores)			
Positive	14.3 (5.9)	15.5 (6.4)	$Z = 2.16$, $p = .029$
Negative	14.3 (7.3)	15.4 (7.2)	$Z = 1.74$, $p = .081$
General	31.9 (9.9)	32.3 (10.0)	$Z = 0.39$, $p = .701$
Total	60.6 (19.7)	63.3 (19.6)	$Z = 1.56$, $p = .117$
Number of recent life events (median, range)	4 (0-10)	3 (0-10)	$Z = -3.90$, $p < .001$
Burden of recent life events (sum)	7.5 (7.0)	5.6 (5.6)	$Z = -3.40$, $p = .001$
CTQ-SF (subscale scores)			
Emotional Abuse	10.8 (4.8)	9.3 (4.1)	$Z = -3.45$, $p < .001$
Physical Abuse	6.6 (3.4)	6.4 (2.6)	$Z = -1.05$, $p = .302$
Sexual Abuse	6.5 (3.4)	5.7 (2.2)	$Z = -3.04$, $p = .002$
Emotional Neglect	11.7 (4.4)	11.6 (3.8)	$Z = -0.25$, $p = .804$
Physical Neglect	7.5 (3.0)	7.5 (2.6)	$Z = 0.01$, $p = 1$
GAF-Disability (past month)	49.6 (14.7)	47.6 (13.4)	$Z = -1.63$, $p = .104$
GAF-Symptoms (past month)	46.9 (13.9)	45.9 (14.2)	$Z = -0.83$, $p = .400$
BDI-II (total score)	25.9 (12.9)	22.1 (12.6)	$Z = -3.21$, $p = .002$

Abbreviations: BDI: Beck Depression Inventory; CTQ-SF: Childhood Trauma Scale-Short Form; GAF: Global Assessment of Functioning; PANSS = Positive and Negative Syndrome Scale; ROP = Recent-Onset Psychosis

Supplementary Table 3. Comparison of baseline demographic and clinical characteristics of those participants included in longitudinal modeling and those participants excluded due to missing data. Means (SD) unless stated otherwise.

Variable	Included (n = 337)	Excluded (n = 210)	Comparison
Studygroup (% ROP)	50.1	53.8	χ^2 = 0.69, *p* = .412
Sex (% female)	46.0	50.0	χ^2 = 0.83, *p* = .386
Age	24.6 (5.6)	24.8 (5.6)	Z = 0.50, *p* = .620
PANSS (subscale scores)			
Positive	15.2 (6.4)	14.6 (6.0)	*Z* = -1.10, *p* = .271
Negative	15.2 (7.0)	14.4 (7.7)	*Z* = -1.24, *p* = .218
General	32.8 (9.6)	31.0 (10.4)	*Z* = -2.09, *p* = .038
Total	63.2 (19.0)	60.0 (20.6)	*Z* = -1.86, *p* = .059
Number of recent life events (median, range)	3 (0-10)	3 (0-10)	*Z* = -1.91, *p* = .060
Burden of recent life events (sum)	6.8 (6.4)	5.9 (6.3)	*Z* = -1.66, *p* = .101
CTQ (subscale scores)			
Emotional Abuse	9.8 (4.3)	10.4 (4.8)	*Z* = 1.28, *p* = .202
Physical Abuse	6.3 (2.8)	6.8 (2.4)	*Z* = 1.51, *p* = .135
Sexual Abuse	6.1 (2.8)	6.0 (3.1)	*Z* = -0.11, *p* = .920
Emotional Neglect	11.4 (4.0)	12.1 (4.3)	*Z* = 1.58, *p* = .115
Physical Neglect	7.3 (2.7)	8.0 (3.0)	*Z* = 2.50, *p* = .011
GAF-Disability (past month)	48.7 (14.3)	48.4 (13.7)	*Z* = -0.25, *p* = .804
GAF-Symptoms (past month)	46.0 (14.0)	47.0 (14.1)	*Z* = 0.76, *p* = .437
BDI (total score)	23.8 (12.2)	24.3 (14.0)	*Z* = 0.42, *p* = .682

Abbreviations: BDI: Beck Depression Inventory; CTQ-SF: Childhood Trauma Scale-Short Form; GAF: Global Assessment of Functioning; PANSS = Positive and Negative Syndrome Scale; ROP = Recent-Onset Psychosis

Supplementary Results

Supplementary Results 1. Robustness analyses.

The CS-coefficient indicated high stability for the edge weights of the network in figure 1a (original network without controlling for covariates), as 75% of the sample could be dropped while maintaining a correlation of at least $r = .7$ with the edge weights of the original network model. The corresponding plot is available in supplementary figure 3. Regarding estimates of individual edges, the bootstrapping analysis suggested that all edges present in the original network were also included in the majority of network models built on bootstrapped samples, and that the edge weights were overall estimated with good accuracy (supplementary figure 2). Overall, we found a similar pattern for the network model when additionally including different types of childhood trauma as covariates (figure 1b). CS-coefficient suggested high stability (CS = 0.75, supplementary figure 5). Edges retained in the original covariate network model were present in the majority of bootstrapped networks, and edge weights were overall estimated with good accuracy (supplementary figure 4).

Supplementary Results 2. Comparison of networks estimated in CHR and ROP.

Statistical network comparison based on permutation tests indicated no significant differences in network structure (Test statistic $M = 0.25$, $p = .075$), global strength (Test statistic $S = 1.31$, $p = .157$) nor any individual edge weights (all p's > .210 after controlling the false discovery rate) between networks estimated in CHR and ROP (for a visualization of the networks, supplementary figure 6).

Supplementary Results 3. Comparison of networks estimated in women and men.

Statistical network comparison based on permutation tests indicated no significant differences in network structure (Test statistic $M = 0.20$, $p = .391$), global strength (Test statistic $S = 1.44$, $p = .110$) nor any individual edge weights (all p's > .240 after controlling the false discovery rate) between networks estimated in women and men (for a visualization of the networks, supplementary figure 7).

Supplementary Figures

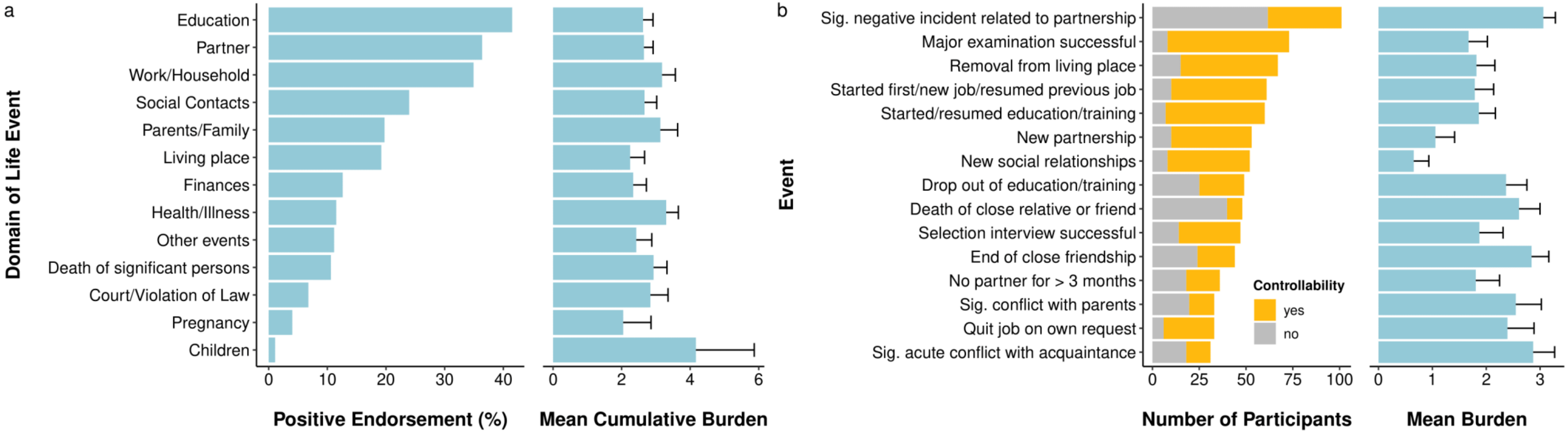

Supplementary Figure 1. Life events in the early psychosis spectrum reported at baseline (*N* = 547). a) Domains of the Cologne Chart of Life Events (CoLE [3]) with rates of positive endorsement and mean cumulative burden. Positive endorsement indicates if a participant reported at least one life event of the respective domain. Mean burden is cumulative as participants could name multiple life events per domain. Life events directly linked to the mental health status of the participants (e.g. hospitalization, start of treatment) were excluded. b) The fifteen most reported individual life events, along with their reported mean burden. Controllability depicts the number of participants that experienced the life event as controllable. Error bars represent the 95% confidence interval.

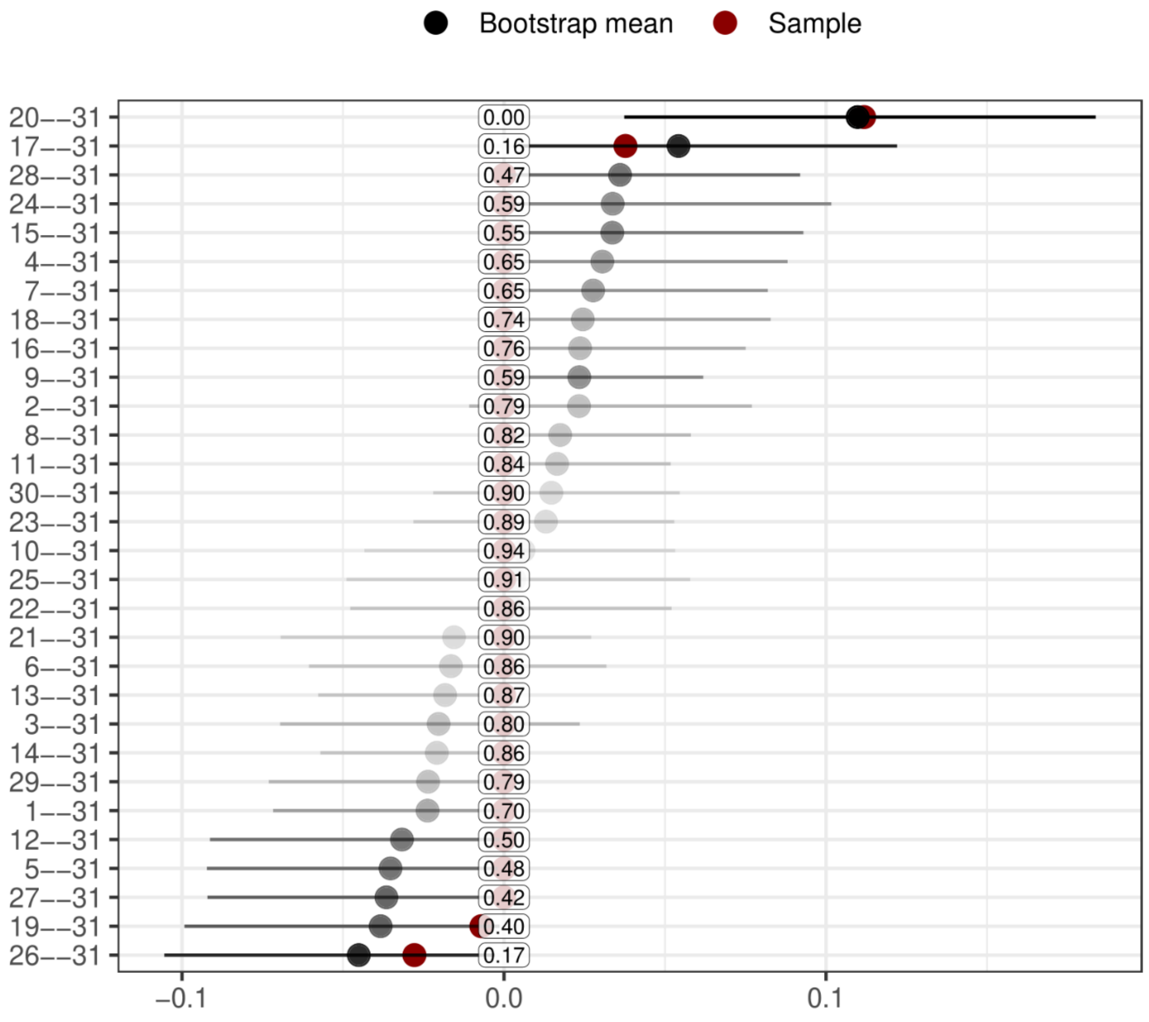

Supplementary Figure 2. Edge values with 95% confidence intervals obtained from bootstrapping in the original sample for the main network model. For readability, we only plot edges related to burden of life events. Confidence intervals are calculated based on those networks in which the edge was included (rather than set to zero). The transparency of the confidence interval reflects how often the edge was included in the networks generated in the bootstrapping procedure. The number in the box gives the proportion of sampled networks in which each edge was set to zero. For the node labels, see figure 1 in the main text.

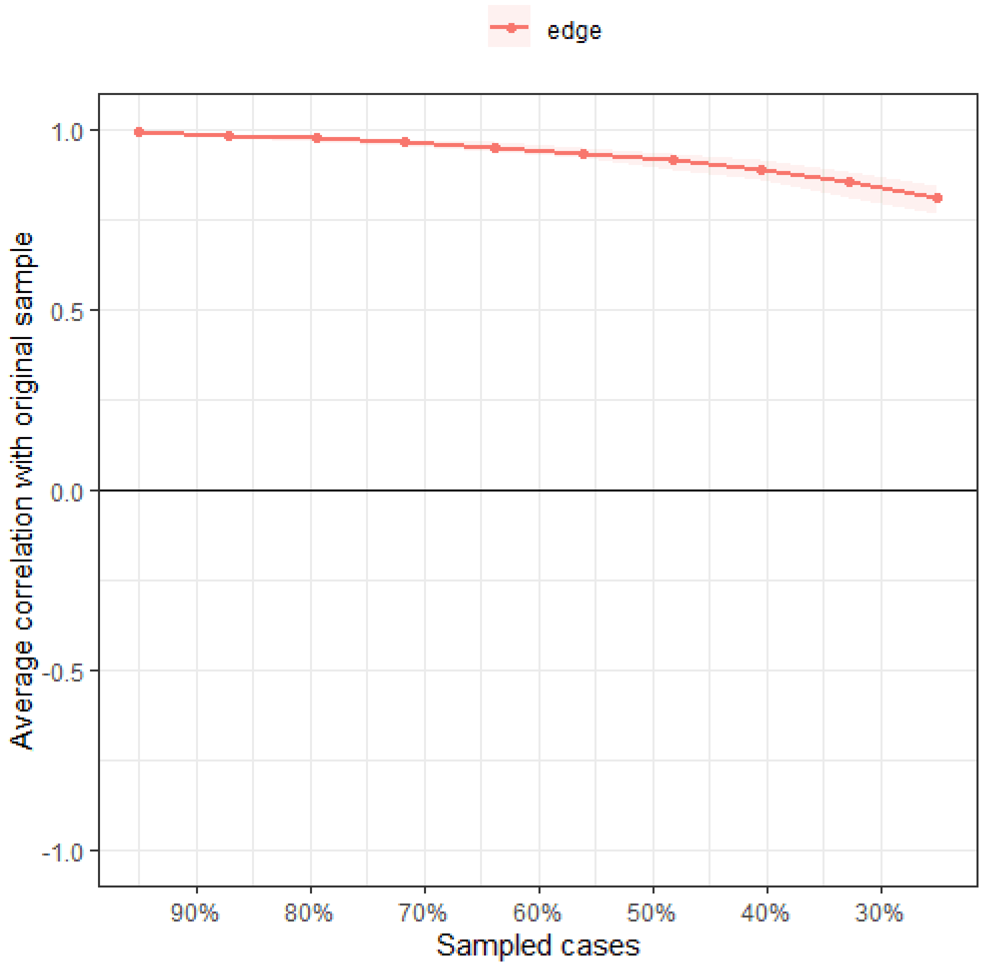

Supplementary Figure 3. Case-dropping bootstrap for the main network model. The x-axis depicts the percentage of cases of the sample used at each step. The y-axis depicts the average of correlations between the edge weights from the original network and the edge weights from networks that were re-estimated after dropping increasing percentages of cases. Lines indicate the means and areas indicate the range from the 2.5th quantile to the 97.5th quantile. The maximum proportion of observations that could be dropped while confidently (95%) retaining results that correlate highly (r > .7) with the edge weights in the original sample was 75%, indicating high stability[1].

.

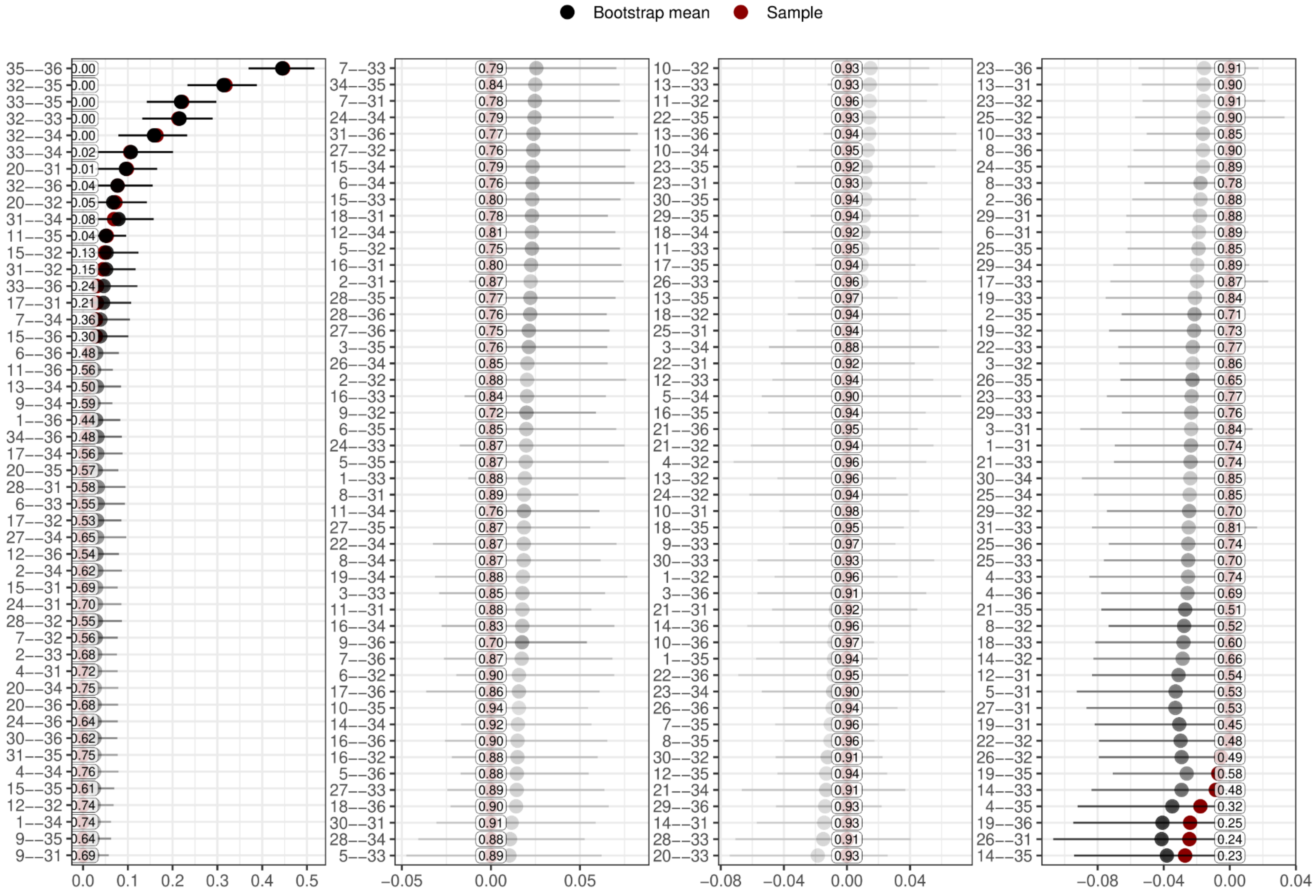

Supplementary Figure 4. Edge values with 95% confidence intervals obtained from bootstrapping for the main network model after inclusion of different childhood trauma types as covariates. For readability, we only plot edges related to life events and the types of childhood trauma. Confidence intervals are calculated based on those networks in which the edge was included (rather than set to zero). The transparency of the confidence interval reflects how often the edge was included in the networks generated in the bootstrapping procedure. The number in the box gives the proportion of sampled networks in which each edge was set to zero. For the node labels, see figure 1 in the main text.

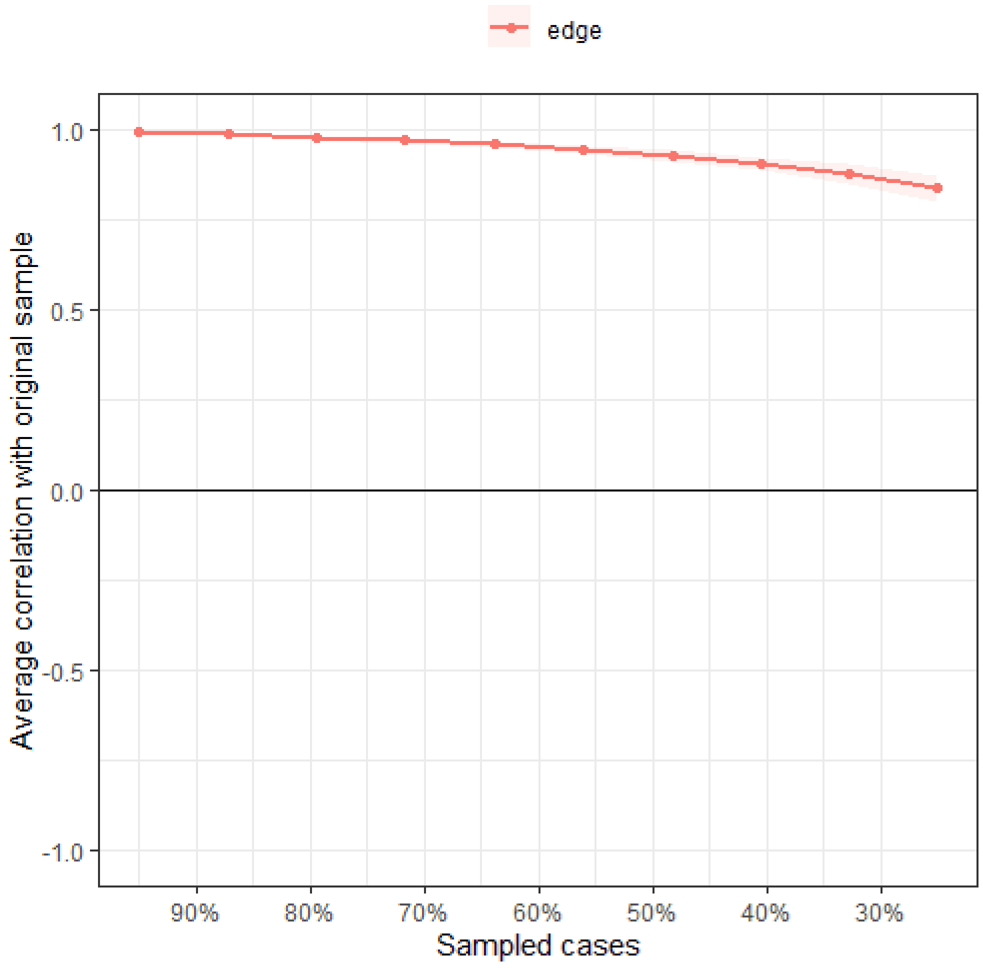

Supplementary Figure 5. Case-dropping bootstrap for the main network model after inclusion of different childhood trauma types as covariates. The x-axis depicts the percentage of cases of the sample used at each step. The y-axis depicts the average of correlations between the edge weights from the original network and the edge weights from networks that were re-estimated after dropping increasing percentages of cases. Lines indicate the means and areas indicate the range from the 2.5th quantile to the 97.5th quantile. The maximum proportion of observations that could be dropped while confidently (95%) retaining results that correlate highly (r > .7) with the edge weights in the original sample was 75%, indicating high stability[1].

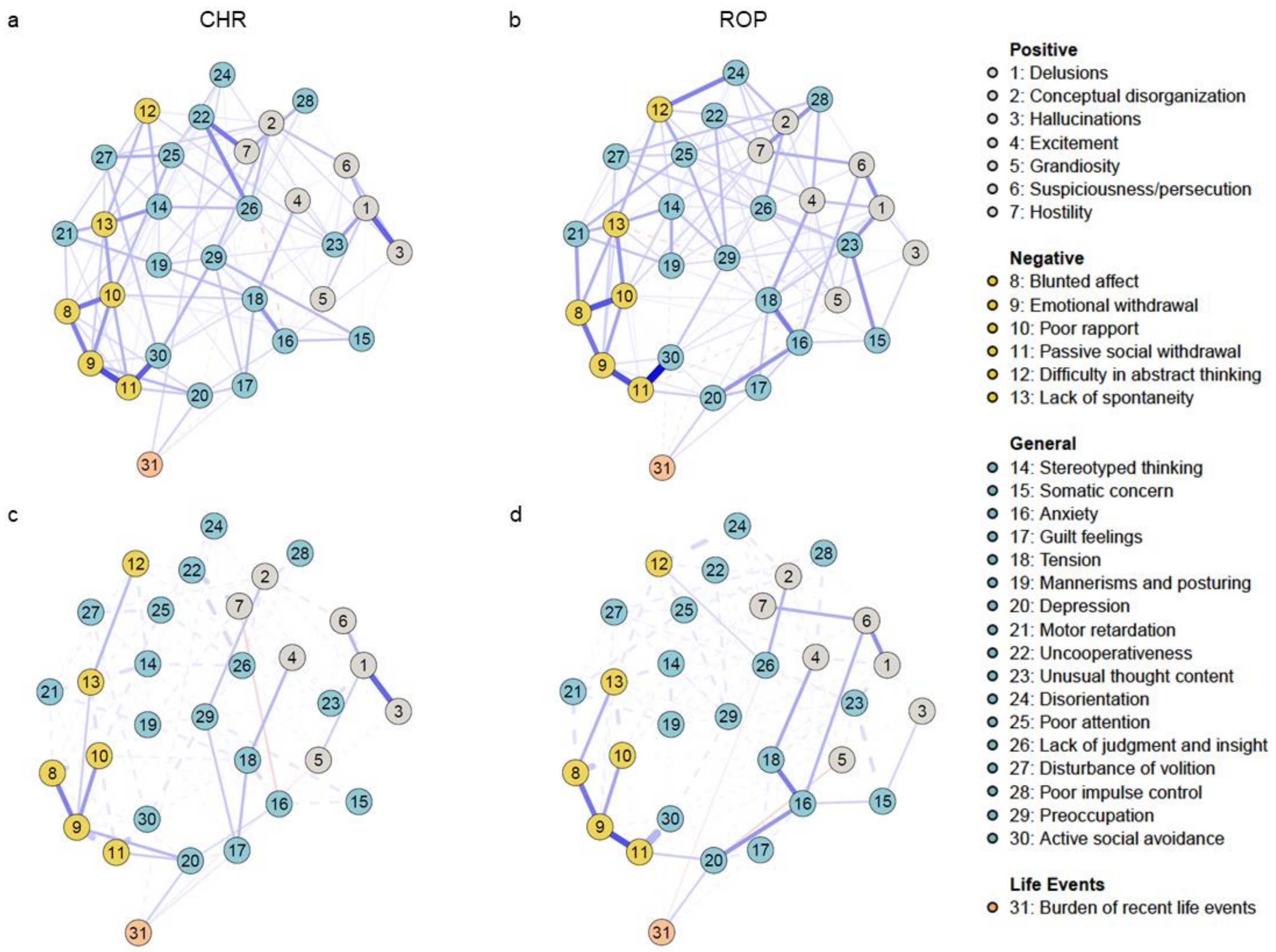

Supplementary Figure 6. Cross-sectional networks of relationships between burden of recent life events and symptomatology assessed with the Positive and Negative Syndrome Scale (PANSS) estimated separately in Clinical High-Risk (CHR) and Recent Onset Psychosis (ROP) participants. Upper panel: Network depicting unique associations between burden of recent life events and individual symptoms a) in CHR and b) in ROP participants. The wider the edge, the stronger the association. Blue (red) edges reflect positive (negative) connections. Lower panel: Networks highlighting shortest paths[2] between burden of recent life events and the positive and negative symptom domain of the PANSS c) in CHR and d) in ROP participants. Solid lines represent shortest paths, dashed lines represent connections that do not lie on the shortest paths. The wider the edge, the stronger the association. Blue (red) edges reflect positive (negative) connections.

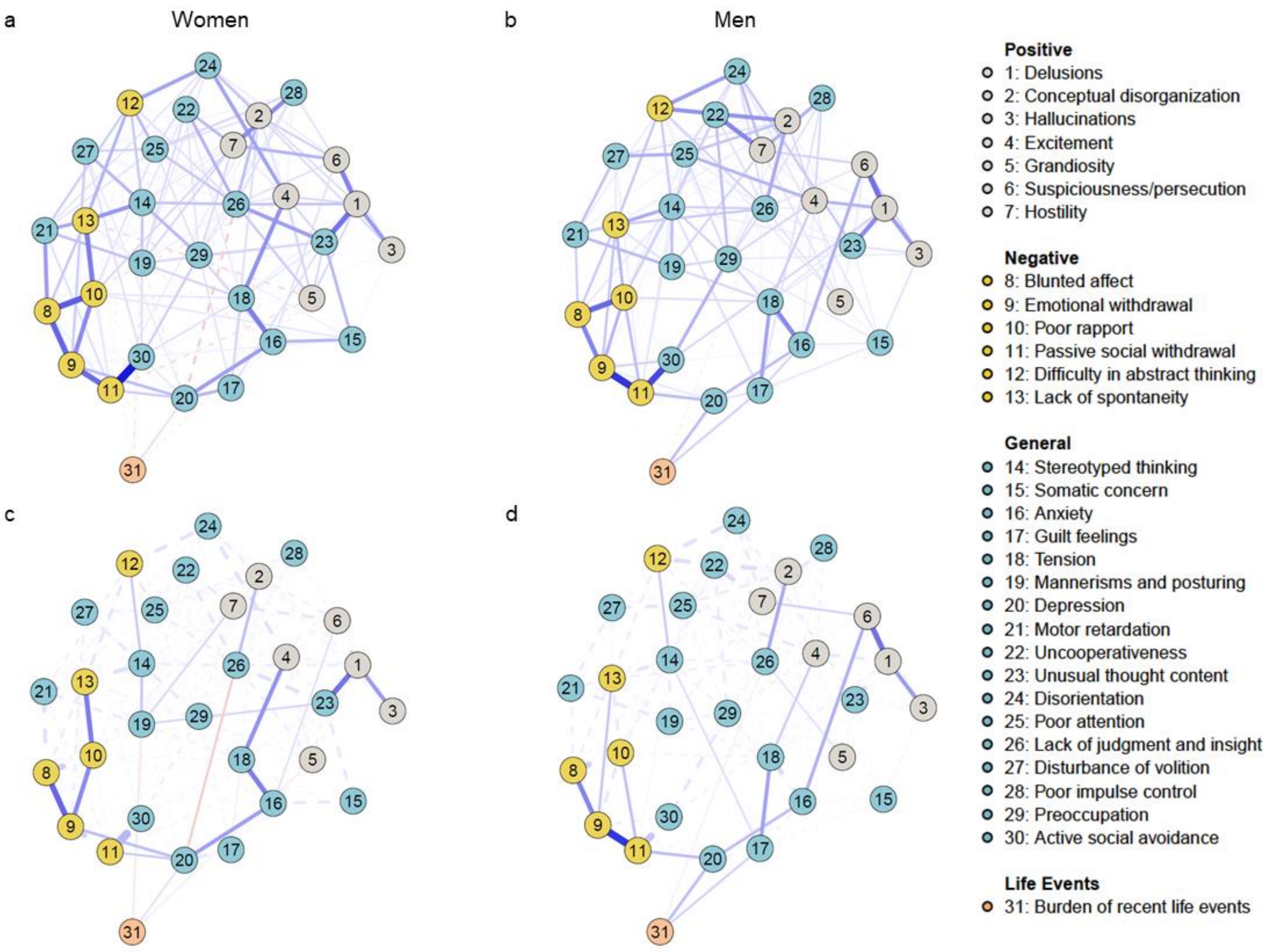

Supplementary Figure 7. Cross-sectional networks of relationships between burden of recent life events and symptomatology assessed with the Positive and Negative Syndrome Scale (PANSS) estimated separately in women and men. Upper panel: Network depicting unique associations between burden of recent life events and individual symptoms a) in women and b) in men. The wider the edge, the stronger the association. Blue (red) edges reflect positive (negative) connections. Lower panel: Networks highlighting shortest paths[2] between burden of recent life events and the positive and negative symptom domain of the PANSS c) in women and d) in men. Solid lines represent shortest paths, dashed lines represent connections that do not lie on the shortest paths. The wider the edge, the stronger the association. Blue (red) edges reflect positive (negative) connections.

Supplementary Figure 8. The Cologne Chart of Life Events. The Cologne Chart of Life Events (CoLE[3]) was adapted from the Munich Life Event List[4] and comprises a list with 117 events from 12 domains:

PRONIA Assessments

FETZ Chart of Life Events

1. In the last ...[1], did you experience any special event concerning...		2. *After collecting life events and their domains, ask following questions:*					3. *Final question!*
A. Education Y N<br>B. Work / Household Y N<br>C. Partner Y N<br>D. Pregnancy Y N<br>E. Children Y N<br>F. Parents / Family Y N<br>G. Social Contacts Y N<br>H. Death Y N<br>I. Home Y N<br>J. Finances Y N<br>K. Court Y N<br>L. Health/Illness Y N<br>M. Other events Y N	**Event** (number according to list on next page)	**How many days ago did it start?**	**Course**<br>1,2,3,... = frequency<br>8 = continuously<br>9 = not known	**Experienced as controllable?**<br>Yes / No	**Subjective evaluation**<br>1 = very positive<br>2 = positive<br>3 = neutral<br>4 = negative<br>5 = very negative	**Subjective burden**<br>0 = no burden<br>1 = slight<br>2 = moderate<br>3 = severe<br>4 = very severe	**Rank order**<br>1 = most important<br>2= second most important,<br>3, 4...

[1] Healthy Controls and Patients: At T0 ask for the last 12 months
Healthy Controls: At T1 and T2 ask for period since the last examination (If T1 examination was skipped ask for the whole period since T0)
Patients: At Follow-up (IV3,6,12,15 and T1/T2) ask for the last 3 months (If the patient did not appear regularly ask for the whole period since the last examination)

© Cologne FETZ

PRONIA LIFE EVENTS INSTRUMENT – Coding

A. Education

1. Selection interview successful
2. Selection interview unsuccessful
3. Started/ resumed an education or vocational training
4. Major[2] examination successful
5. Major examination unsuccessful
6. Drop out of education / training
7. Acute significant conflicts with other students
8. Long-standing conflicts with students/teachers (> 3 months)
9. Significant positive change of conditions at place of education / training [3]
10. Significant negative change of conditions at place of education / training (see footnote 2)
11. (1) to (6) happened to a close relative / close friend (if yes, please specify relationship)

B. Work / Household

12. Selection interview successful
13. Selection interview unsuccessful
14. Started first/new job / resumed previous job (after > 6 months)
15. Quit job on own request
16. Dismissed
17. Acute significant conflicts with colleagues/boss
18. Long-standing conflicts with colleagues/boss (> 3 months)
19. Significant positive change of conditions at work[4]
20. Significant negative change of conditions at work (see footnote 3)
21. Significant professional success
22. Significant professional failure
23. unable to work (> 3 months)
24. unemployed (> 3 months)
25. Long-standing overwhelming due to job/household related tasks (> 3 months)
26. Military / voluntary service started/resumed/finished
27. Early retirement
28. Any significant events according to the list above happening to a close relative / close friend (if yes, please specify relationship)

If not the principle earner:

29. Significant professional success of principal earner
30. Significant professional failure of principal earner
31. Unemployment / other reasons for diminished income[5] of principal earner

C. Partner

32. New partnership (> 3 months)
33. First sexual intercourse
34. Significant negative incident related to partnership[6], including failure to establish new partnership with a person known for > 3 months
35. Significant positive incident (including marriage, decision to cohabit)
36. Significant long-standing conflict with partner (> 3 months)
37. No partner for > 3 months
38. Any significant positive event happening to partner
39. Any significant negative event happening to partner

D. Pregnancy

40. Infertility
41. Pregnancy
42. Pre/postnatal complications
43. Miscarriage
44. Termination of pregnancy
45. Birth
46. Stillbirth
47. Sterilization
48. Any of the events above happened to partner
49. Any of the events above happened to close relative /close friend

E. Children

50. Moving out / in again
51. Any negative acute change in relationship to children
52. Any longstanding conflict with children with impact on relationship (> 3 months)
53. Any significant physical or mental health problems of children[7]
54. Conflict with law / becoming criminal
55. Acute adverse events (e.g. victim of significant violence)

---

[2] major: examination has special meaning for training, i.e. required to proceed or final exams
[3] e.g. change of school or class
[4] e.g. significant impact change of usual working conditions, i.e. procedures or tasks
[5] e.g. unable to work due to illness
[6] Separation, divorce, adultery of partner/respondent, significant crisis due to other reasons
[7] [experienced as] life threatening, leading to disability, hospitalization, drug abuse etc.

© Cologne FETZ

56. Long-standing adverse events (e.g. bullying)
57. Marriage Separation/Divorce

F. Parents / Family

58. Moving out of parent's home
59. Moving back to parents (< 6 months after leaving)
60. Significant conflict with parents
61. Significant conflict with close relatives living in the same household
62. Significant conflict with close relatives living outside household
63. Significant long-standing conflict with parents
64. Significant long-standing conflict with close relatives living in the same household
65. Significant long-standing conflict with close relatives living outside household
66. Significant conflict of parents
67. Separation / divorce of parents

G. Social Contacts

68. New social relationships (> 3 months, not partnership)
69. End of close friendship
70. Significant acute conflict with acquaintance
71. Long-standing conflict with acquaintance
72. Loneliness (> 3 months)

H. Death of personally significant persons

73. Partner
74. Child
75. Parent
76. Close relative or close friend
77. Other personally significant person

I. Living place

78. Removal
79. Building house
80. Major refurbishment / conversion
81. Moving to a favorable neighborhood (save, good relationships)
82. Moving to an adverse neighborhood (dangerous, violent, criminality)
83. Significant acute conflict with neighbors
84. Significant long-standing conflict with neighbors (> 3 months)
85. Contract was terminated by owner
86. Becoming homeless
87. Any significant events according to the list above happening to a close relative / close friend (if yes, please specify relationship)

J. Finances

88. Significant financial problems
89. Significant improvement of financial conditions
90. Significant worsening of financial conditions
91. Any significant events according to the list above happening to a close relative / close friend (if yes, please specify relationship)

K. Court/Violation of Law

92. Criminal offense against person
93. Criminal offense against person's properties
94. Committed crime
95. Prosecuted
96. Contact to police (as a suspect)
97. Detention
98. Imprisoned / brought to corresponding institution (not hospital)
99. Fine or corresponding penalty (not prison or corresponding institution)
100. Any significant events according to the list above happening to a close relative / close friend (if yes, please specify relationship)

L. Health/Illness

101. Accident with personal damage
102. Hospitalization (incl. day time clinic)
103. Surgery
104. Significant somatic illness (requiring continuous treatment or disabling)
105. Significant mental Illness (requiring continuous treatment or disabling)
106. Suicide attempt
107. Discharge from hospital / day time clinic
108. Starting pharmacological treatment
109. Starting psychological consultation / treatment
110. Any significant events according to the list above happening to a close relative / close friend (if yes, please specify relationship)

M. Other events

111. Accident (no personal damage)
112. Disaster victim (fire, hurricane etc.)
113. Unwanted reduction / cessation of personally significant leisure time activities (sports, music, travelling etc.)
114. Getting reminded of traumatic events
115. Getting a pet
116. Losing a pet (if significant relationship)
117. Any significant events according to the list above happening to a close relative / close friend (if yes, please specify relationship)

© Cologne FETZ

Supplementary References

1. Epskamp, S., Borsboom, D. & Fried, E. I. Estimating psychological networks and their accuracy: A tutorial paper. *Behav. Res. Methods* **50**, 195–212 (2018).

2. Brandes, U. On variants of shortest-path betweenness centrality and their generic computation. *Soc. Networks* **30**, 136–145 (2008).

3. Ruhrmann, S. *The Cologne Chart of Life Events (CoLE)*. (2013).

4. Wittchen, H.-U., Ahmoi Essau, C., Hecht, H., Teder, W. & Pfister, H. Reliability of life event assessments: test—retest reliability and fall-off effects of the Munich interview for the assessment of life events and conditions. *J. Affect. Disord.* **16**, 77–91 (1989).

PRONIA consortium members

PRONIA consortium members listed here performed the screening, recruitment, rating, examination, and follow-up of the study participants and were involved in implementing the examination protocols of the study, setting up its information technological infrastructure, and organizing the flow and quality control of the data analyzed in this article between the local study sites and the central study database:

Mark Sen Dong [2], Anne Erkens [2], Eva Gussmann [2], Shalaila Haas [2], Alkomiet Hasan [2], Claudius Hoff [2], Ifrah Khanyaree [2], Aylin Melo [2], Susanna Muckenhuber-Sternbauer [2], Janis Kohler [2], Ömer Faruk Özturk [2], David Popovic [2], Adrian Rangnick [2], Sebastian von Saldern [2], Rachele Sanfelici [2], Moritz Spangemacher [2], Ana Tupac [2], Maria Fernanda Urquijo [2], Johanna Weiske [2], Antonia Wosgien [2], Karsten Blume [1], Mauro Seves [1], Nathalie Kaiser [1], Tanja Pilgram [1], Thorsten Lichtenstein [1], Christiane Woopen [1], Christina Andreou [8], Laura Egloff [8], Fabienne Harrisberger [8], Claudia Lenz [8], Letizia Leanza [8], Amatya Mackintosh [8], Renata Smieskova [8], Erich Studerus [8], Anna Walter [8], Sonja Widmayer [8], Chris Day [3], Sian Lowri Griffiths [3], Mariam Iqbal [3], Mirabel Pelton [3], Pavan Mallikarjun [3], Ashleigh Lin [3], Alexander Denissoff [14], Anu Ellila [14], Tiina From [14], Markus Heinimaa [14], Tuula Ilonen [14], Paivi Jalo [14], Heikki Laurikainen [14], Maarit Lehtinen [14], Antti Luutonen [14], Akseli Makela [14], Janina Paju [14], Henri Pesonen [14], Reetta-Liina Armio (Saila) [14], Elina Sormunen [14], Anna Toivonen [14], Otto Turtonen [14], Carlo Altamura [10], Marika Belleri [10], Francesca Bottinelli [10], Adele Ferro [10], Marta Re [10], Emiliano Monzani [16], Mauro Percudani [16], Maurizio Sberna [16], Armando D'Agostino [17], Lorenzo Del Fabro [17], Giampaolo Perna [18], Maria Nobile [18], Alessandra Alciati [18], Matteo Balestrieri [19], Carolina Bonivento [19], Giuseppe Cabras [19], Franco Fabbro [19], Marco Garzitto [20], Sara Piccin [20], Giuseppe Blasi [7], Linda A. Antonucci [7], Giulio Pergola [7], Grazia Caforio [7], Leonardo Faio [7], Tiziana Quarto [7], Barbara Gelao [7], Raffaella Romano [7], Ileana Andriola [7], Andrea Falsetti [7], Marina Barone [7], Roberta Passatiore [7], Marina Sangiuliano [7], Marian Surman [11], Olga Bienek [11], Georg Romer [11], Udo Dannlowski [11], Christian Schmidt-Kraepelin [13], Susanne Neufang [13], Alexandra Korda [13], Henrik Rohner [13], Ana Beatriz Solana [21], Manuela Abraham [21], Nicolas Hehn [21], Timo Schirmer [21].

Institutional affiliations:

[16] Programma 2000, Niguarda Hospital, Milan, Italy

[17] San Paolo Hospital, Milan, Italy

[18] Villa San Benedetto Menni, Albese con Cassano, Italy

[19] Department of Medical Area, University of Udine, Udine, Italy

[20] IRCCS Scientific Institute "E. Medea", Polo FVG, Udine, Italy

[21] General Electric Global Research Inc., Munich, Germany
