# Supplementary material for: General psychopathology links burden of recent life events and psychotic symptoms in a network approach
Source: NPJ Schizophr. 2020 Dec 15;6:40. doi: 10.1038/s41537-020-00129-w (PMC7738498; doi:10.1038/s41537-020-00129-w)
Supplement: Supplementary file 2 — Reporting Summary Checklist [file 41537_2020_129_MOESM2_ESM.pdf]

nature research

Corresponding author(s): Linda T. Betz

Last updated by author(s): Aug 10, 2020

Reporting Summary

Nature Research wishes to improve the reproducibility of the work that we publish. This form provides structure for consistency and transparency in reporting. For further information on Nature Research policies, see our Editorial Policies and the Editorial Policy Checklist.

Statistics

For all statistical analyses, confirm that the following items are present in the figure legend, table legend, main text, or Methods section.

n/a	Confirmed	
☐	☒	The exact sample size (*n*) for each experimental group/condition, given as a discrete number and unit of measurement
☐	☒	A statement on whether measurements were taken from distinct samples or whether the same sample was measured repeatedly
☐	☒	The statistical test(s) used AND whether they are one- or two-sided<br>*Only common tests should be described solely by name; describe more complex techniques in the Methods section.*
☐	☒	A description of all covariates tested
☒	☐	A description of any assumptions or corrections, such as tests of normality and adjustment for multiple comparisons
☐	☒	A full description of the statistical parameters including central tendency (e.g. means) or other basic estimates (e.g. regression coefficient) AND variation (e.g. standard deviation) or associated estimates of uncertainty (e.g. confidence intervals)
☐	☒	For null hypothesis testing, the test statistic (e.g. F, t, r) with confidence intervals, effect sizes, degrees of freedom and P value noted<br>*Give P values as exact values whenever suitable.*
☒	☐	For Bayesian analysis, information on the choice of priors and Markov chain Monte Carlo settings
☒	☐	For hierarchical and complex designs, identification of the appropriate level for tests and full reporting of outcomes
☒	☐	Estimates of effect sizes (e.g. Cohen's *d*, Pearson's *r*), indicating how they were calculated

Our web collection on statistics for biologists contains articles on many of the points above.

Software and code

Policy information about availability of computer code

Data collection	No software was used in data collection.
Data analysis	For all aspects of the data analysis, we used the "R" language for statistical computing, version 3.6.3. Beyond the functionalities implemented in base R, we used the packages "coin", "qgraph", "NetworkComparisonTest", "bootnet", and "psychonetrics".

For manuscripts utilizing custom algorithms or software that are central to the research but not yet described in published literature, software must be made available to editors and reviewers. We strongly encourage code deposition in a community repository (e.g. GitHub). See the Nature Research guidelines for submitting code & software for further information.

Data

Policy information about availability of data

All manuscripts must include a data availability statement. This statement should provide the following information, where applicable:

- Accession codes, unique identifiers, or web links for publicly available datasets
- A list of figures that have associated raw data
- A description of any restrictions on data availability

The data are not publicly available due to privacy restrictions.

Field-specific reporting

Please select the one below that is the best fit for your research. If you are not sure, read the appropriate sections before making your selection.

☒ Life sciences ☐ Behavioural & social sciences ☐ Ecological, evolutionary & environmental sciences

For a reference copy of the document with all sections, see nature.com/documents/nr-reporting-summary-flat.pdf

Life sciences study design

All studies must disclose on these points even when the disclosure is negative.

Sample size	Given the observational design of the study, no a priori sample size calculation was performed. We conducted several robustness analyses based on different bootstrapping procedures that indicated that sample size was sufficient in the context of established guidelines in the field of network analysis (see doi:10.3758/s13428-017-0862-1).
Data exclusions	No data were excluded from the study, except when required scientifically important information for the analysis (i.e., burden of life events, symptom scores) were not available.
Replication	Not applicable. Observational, non-experimental study.
Randomization	Not applicable. Observational study.
Blinding	Not applicable. Observational study.

Reporting for specific materials, systems and methods

We require information from authors about some types of materials, experimental systems and methods used in many studies. Here, indicate whether each material, system or method listed is relevant to your study. If you are not sure if a list item applies to your research, read the appropriate section before selecting a response.

Materials & experimental systems

n/a	Involved in the study	
☒	☐	Antibodies
☒	☐	Eukaryotic cell lines
☒	☐	Palaeontology and archaeology
☒	☐	Animals and other organisms
☐	☒	Human research participants
☒	☐	Clinical data
☒	☐	Dual use research of concern

Methods

n/a	Involved in the study	
☒	☐	ChIP-seq
☒	☐	Flow cytometry
☒	☐	MRI-based neuroimaging

Human research participants

Policy information about studies involving human research participants

Population characteristics	Patients (15-40 years) with clinical high risk for psychosis and recent onset psychosis from both in- and outpatient settings.
Recruitment	Participants were recruited in 10 academic early-recognition services in 5 European countries, i.e., Finland, Germany, Italy, Switzerland, and the United Kingdom between February 2014 and December 2017.
Ethics oversight	The study was registered at the German Clinical Trials Register (identifier DRKS00005042) and approved by the local research ethics committees in each of the 10 locations.

Note that full information on the approval of the study protocol must also be provided in the manuscript.
